# The frequency of, and adherence to, single maintenance and reliever therapy instructions in asthma: a descriptive analysis

**DOI:** 10.1038/npjpcrm.2016.38

**Published:** 2016-07-21

**Authors:** Rachael L DiSantostefano, Nada Boudiaf, David A Stempel, Neil C Barnes, Andrew P Greening

**Affiliations:** 1 Department of Epidemiology, PAREXEL International, RTP, NC, USA; 2 Worldwide Epidemiology, GSK, Uxbridge, UK; 3 Respiratory R&D, GSK, RTP, NC, USA; 4 Respiratory R&D, GSK, Uxbridge, UK

## Abstract

Inhaled corticosteroid/long-acting β_2_-agonist (ICS/LABA) fixed-dose combinations are recommended regular maintenance options for asthma. ICS/LABAs containing formoterol may also be indicated for single maintenance and reliever therapy (SMART). This analysis evaluated the frequency of SMART dosing of budesonide/formoterol fixed-dose combination (BFC) in the United Kingdom. Secondary objectives were to assess adherence and use of short-acting ß_2_-agonists (SABAs). This was a descriptive analysis of treatment patterns using the UK Clinical Practice Research Datalink-GP OnLine Database data (2009–2013). SMART dosing was determined when prescription instructions contained guidance for daily dosing plus ‘and when required’. Treatment and prescription refill patterns of BFC and SABA were described in the year following the index date to identify adherence and SMART dosing instructions versus other dosing regimens. Of 14,818 patients identified, 173 (1.2%) had evidence of prescriptions for SMART dosing at their index BFC prescription. Despite being prescribed SMART dosing, 91 of 173 patients (53%) were additionally dispensed SABA in the year following the index date. The mean number of BFC inhalers used was less than required for daily treatment for SMART and non-SMART dosing groups (4.7 and 4.8, respectively).This analysis suggests that SMART dosing is infrequent when examining dosing instructions. Therefore, results of randomised clinical trials using SMART dosing may not translate to clinical practice in the United Kingdom because of the low level of SMART prescription, concurrent use of SABA, and inadequate refill persistence observed. Further research is needed to understand SMART dosing in real-world clinical practice.

## Introduction

Inhaled corticosteroid/long-acting β-agonist (ICS/LABA) fixed-dose combination medications are the recommended treatment for patients with asthma not adequately controlled on an ICS alone.^1,2^ All ICS/LABA treatments are indicated for a consistent daily dose of once or twice a day. Some ICS/LABA treatments for asthma containing formoterol (a LABA) may also be indicated for use as a single maintenance and reliever therapy (SMART), also referred to as single inhaler therapy, where the same combination treatment inhaler is used for both maintenance therapy and relief of symptoms.^1,2^

In randomised clinical trials (RCTs),^3–9^ SMART dosing with budesonide/formoterol fixed-dose combination (BFC) has produced results that demonstrate a reduction in exacerbations requiring oral corticosteroids compared with higher-dose ICS alone or ICS combined with LABA plus as-required short-acting ß_2_-agonists (SABA). However, reviews of these trials highlight that, despite the reduction in exacerbations reported, asthma control was frequently not attained.^10,11^ Although there is evidence from RCTs on the benefits of SMART, there is little real-world evidence on the effectiveness of SMART in the treatment of asthma. The complementary value of real-world data to RCTs has been discussed.^12^

Concerns have also been raised with regard to the understanding and application of the SMART dosing strategy in clinical practice.^11^ There is a potential risk that patients may use their SMART inhaler solely as required rather than adhere to the regular twice-daily maintenance treatment element of SMART dosing. Alternatively, patients may apply the SMART dosing regimen but use additional SABA rescue medication. Thus, SMART dosing requires patient education on appropriate application of the regimen.^2^

A pharmacy audit conducted in 51 branches of a large UK pharmacy multiple by Boyter *et al.*^13^ analysed BFC prescribing in asthma by extracting data from 2484 pharmacy patient medication records. This analysis showed that the use of the SMART strategy for asthma in the United Kingdom was uncommon, with 5.6% (140/2484) prescribed BFC in SMART dosing, which lowered to 2.8% (70/2484) when prescribed SMART according to the UK Summary of Product Characteristics^14^ (appropriate strength, twice-daily and as-needed additional daily inhalations up to defined daily maxima). Adherence to SMART dosing was not as expected, as the number of inhalers ordered was less than that required for the maintenance component of SMART. Moreover, co-prescription of a SABA with SMART was frequent, even though a separate SABA inhaler is not recommended with SMART dosing.^14^

This analysis was performed to gain further insights into SMART dosing in asthma patients in the United Kingdom in a larger sample of patients than used by Boyter *et al*^13^. Specifically, we aimed to investigate whether SMART dosing with BFC was prescribed in real-world clinical practice, whether adherence with maintenance treatment was achieved, and to determine the frequency of SABA dispensed.

## Results

**Figure 1:**
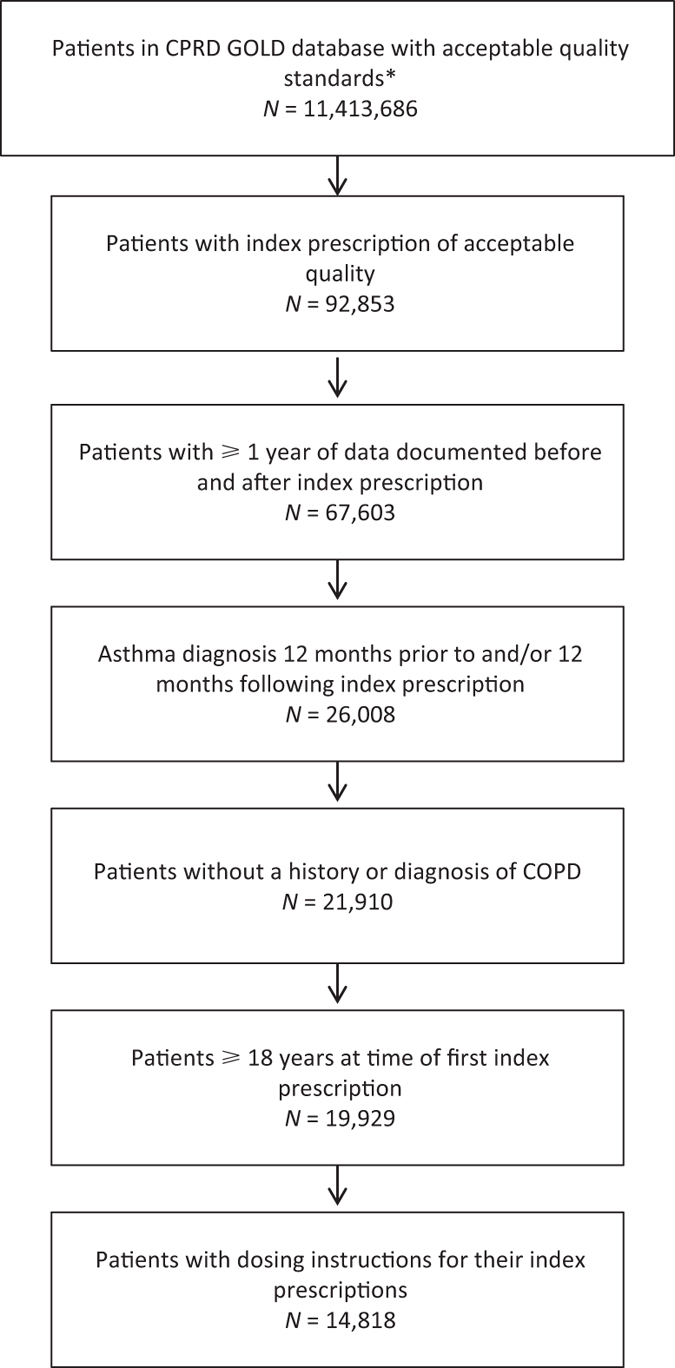
Flow diagram of the patient inclusion process for this analysis. Footnotes: * as defined by CPRD-GOLD. BFC, budesonide formoterol fixed-dose combination; COPD, chronic obstructive pulmonary disease.

The patient inclusion processes for this analysis are shown in Figure 1. The final analysis population included 14, 818 patients with asthma who were prescribed BFC in the period 2009–2013, with at least 1 year of the data before and after their index prescription. The distribution of age and gender was similar for all patients in the analysis, for any dosing strategy (Table 1).

### Frequency of SMART dosing

There were 121,799 prescriptions for BFC issued between 2009 and 2013, with 1,154 (0.9%) determined as SMART (Table 2). Over the 4 years, prescriptions for SMART dosing ranged from 0.7% in 2009 to 1.2% in 2013 (Table 2). Of the total number of patients (*n*=14,818), there were 173 (1.2%) unique patients prescribed BFC with documented SMART dosing instructions (Table 2). There were a further 31 patients with prescription instructions that included both SMART and maintenance dosing instructions; therefore, up to 1.4% of patients had some evidence of SMART dosing (204 of 14,818; data for these additional 31 patients were not further considered, as we focused on the index prescription).

### Additional use of SABA rescue medication

For patients prescribed BFC with SMART dosing, 53% (91 of 173) were additionally prescribed a SABA in the year post index, whereas 82% (11,968 of 14,645) of patients with maintenance dosing were prescribed a SABA (Table 3). A SABA was prescribed on the date of the BFC prescription for 30% (27 of 91) of patients prescribed SMART dosing and 52% (6,188 of 11,968) of patients prescribed maintenance dosing regimens. Among patients with SABA prescriptions, the mean number of SABA canisters prescribed was similar for SMART and maintenance dosing groups (5.7 versus 5.5, respectively; Table 3). In the year following the index BFC prescription, the mean number of BFC inhalers used was similar for SMART dosing and maintenance dosing instructions (mean: 4.7 versus 4.8, respectively; Table 3).

## Discussion

### Main findings

The SMART dosing regimen uses a single device containing ICS/LABA to provide both regular maintenance and rescue medication. Although SMART prescribing with BFC is described in international and some national asthma management guidelines (GINA 2014; BTS-SIGN 2014), there are few data on how frequently it is prescribed in real-world clinical practice. The current analysis reports the documented frequency of SMART dosing in UK clinical practice using prescribing data acquired from a GP database over a 4-year period. The Clinical Practice Research Datalink-GP OnLine Database (CPRD-GOLD) had 14,818 patients with dosing instructions for BFC on their index prescription. The analysis showed that few patients (1.2%) had documented SMART dosing instructions on their index prescription, increasing to 1.4% when subsequent prescriptions were included. Just over half the patients were additionally prescribed a SABA with SMART dosing, contrary to the SMART dosing regimen. Among patients with SABA prescriptions, the mean number of SABA canisters prescribed in the year following the index prescription was similar for SMART and maintenance dosing groups (5.7 versus 5.5, respectively).

Low adherence with daily maintenance ICS/LABA is a frequent issue in clinical practice.^15^ In this analysis, there was also no difference in adherence in BFC persistence among patients with SMART versus maintenance dosing strategy, as measured by the number of prescriptions in the 1-year follow-up period. Refill persistence with both SMART and maintenance dosing strategy was 4.7 inhalers per year and for maintenance dosing it was 4.8 inhalers per year, which is <50% of BFC dispensing required for adequate dosing for the year. This analysis did not assess daily patterns of use to discern whether the medications were used differently depending on dosing instructions. Further, we were unable to use adherence measures (proportion of days covered and medication possession ratio) to compare SMART versus maintenance dosing regimens as planned, as the number of days supplied required for the calculations was missing for most patients, and could not be assumed to be 30 days per inhaler, particularly under a SMART dosing strategy.

### Interpretation of findings in relation to previously published work

Similarly low numbers were reported by Boyter *et al.*,^13^ with 5.6% of patients taking BFC twice daily and when required (i.e., SMART dosing), but this decreased to 2.8% when limiting SMART dosing to that defined by the BFC UK Summary of Product Characteristics^14^ (approved doses and without co-prescription of rescue medication). Co-prescription of SABA was reported in 61 of 140 patients (43%) prescribed SMART dosing compared with 91 of 173 patients (53%) reported in the present analysis. Adherence results also showed that patients in both SMART and maintenance dosing groups had inadequate medication dispensed to support daily maintenance dosing. Boyter *et al*^13^ were concerned that the estimate of SMART dosing might reflect unclear dosing instructions rather than solely infrequent use of SMART dosing. The authors of the present study share these concerns, as well as the lack of documentation in the prescription record demonstrating potential inadequate patient education for this regimen.

There are a number of possible explanations for the low identification of BFC SMART prescribing in the United Kingdom when examining dosing instructions. Perhaps there is a lack of familiarity with SMART among primary care physicians, or physicians are explaining the SMART dosing regimen verbally and are not formally writing the prescription as SMART dosing. Perhaps some drop-down menus in electronic medical records lack the ability to specify SMART dosing.

### Strengths and limitations of this study

This study contributes to the current limited knowledge of the use of SMART dosing in the real-world clinical setting, and it provides results from a second, larger analysis to that performed previously,^13^ with similar results. This study used a general practice database in the United Kingdom to estimate the extent of SMART dosing in clinical practice based on dosing instructions. This was a descriptive study to examine the prevalence of SMART dosing in an unadjusted analysis, and consequently there are limitations.

The primary limitation of our analysis was the potential for ascertainment bias that could have led to an underestimation of SMART prescribing through an imprecise identification of SMART dosing instructions or health-care providers providing verbal prescribing information not included in the written prescribing instructions. A related limitation of this study is that only the top 100,000 free text dosing instructions in the CPRD-GOLD are provided for analysis. In this analysis, one-quarter of the dosing instructions for BFC were unavailable, as they were outside the top 100,000 free text fields provided. It is possible that some of these BFC records excluded from our analysis for missing dosing instructions may have included some SMART dosing, although it is unlikely that the rate would be higher than for the BFC prescriptions analysed. The magnitude of this potential misclassification of SMART dosing is not possible to quantify from our analysis. Furthermore, clinicians may have given verbal instructions as to how to use the SMART approach, which would not have been recorded in the written prescriptions. However, even if this occurred, overall adherence would still have been low. Another limitation of the study design is that we did not examine treatment outcomes, which was outside the scope of the objectives. In addition, the data available cannot be used to ascertain actual patient behaviours (actual use of medications, inhaler technique etc.) that could affect outcomes such as asthma control.

We did not adjust for asthma severity before the index BFC prescription in this descriptive analysis, which could confound comparisons made between usual and SMART dosing groups if there are differences in asthma severity between these groups. For example, if patients with SMART dosing are more mild in terms of asthma severity, this could explain similar use of BFC despite SMART dosing where additional BFC use may be expected. Finally, another limitation is that we have not included beclomethasone dipropionate/formoterol (Fostair, Chiesi, Italy), and this was because Fostair was only available for a short time at the end of the study period. Despite these limitations, this study provides important information about the extent of SMART dosing in the primary care setting.

### Implications for future research, policy and practice

One of the questions with the SMART dosing regimen is whether the benefits of SMART dosing demonstrated in clinical trials translate into clinical practice based on how SMART dosing might be implemented. SMART dosing requires patient understanding of the regimen to include regular maintenance medication and BFC ‘as needed’ for symptoms instead of SABA. A recent study demonstrated that when used ‘as needed’ only, BFC was less effective than BFC treatment taken as regular maintenance therapy when examining time to first treatment failure of the first quarter of patients (11.9 versus 28 weeks).^16^ If patients implement SMART dosing ‘as needed’ instead of maintenance therapy and as needed for symptoms, they may experience earlier treatment failure. This study among the clear users of SMART dosing highlights the need for concern of the risk due to inadequate controller, demonstrated by their persistent need for SABA in addition to BFC rescue.

Thus, when prescribing BFC via SMART dosing instructions, this analysis suggests that there are several key things that should be considered: (1) there needs to be clear dosing instructions available for both maintenance and SMART; (2) there needs to be detailed instructions on when additional or reliever use of the ICS/LABA is required; and (3) there needs to be education that the ICS/LABA is to be used in place of the SABA, and finally instructions on when to contact the health-care professional if worsening symptoms present, which do not respond to treatment. If there is need for refill of SABA or infrequent refill of the BFC, then a review should be made to determine asthma control.

### Conclusions

The SMART dosing regimen uses a single device containing ICS/LABA to provide both regular maintenance and rescue medication. This analysis and the prior analysis^13^ suggest that the findings of the SMART RCTs may not be translatable to clinical practice because of infrequent SMART dosing instructions and low refill persistence noted in these reports. Although RCTs unarguably remain the core of evidence-based medicine, contributions from real-world studies can add to the body of evidence on which clinical decisions are to be based.^12^ More research is needed to understand the use of SMART dosing and its benefits in clinical practice.

## Materials and methods

### Analysis design

This was a descriptive analysis of dosing instructions for BFC therapy (Symbicort Turbohaler, Astra Zeneca AB, Södertälje, Sweden) prescribed to patients with asthma. The analysis used data from the CPRD-GOLD, a primary care database in the United Kingdom (www.cprd.com). Ethics approval was obtained from the Independent Scientific Advisory Committee, which oversees research in CPRD: protocol 14_045.

### Study population

The database was used to identify patients with ⩾1 BFC prescription between the 2009 and 2013 calendar years; ⩾1-year (365 days) data available in the database before and after their first BFC prescription; a diagnosis of asthma 1 year before and/or after their index BFC prescription; and who were ⩾18 years of age at the time of first BFC prescription. Patients were selected for this analysis if their prescription met the acceptable quality according to CPRD standards (e.g., checks of continuity, completion in accordance to CPRD recording standards). Any patients with a history or diagnosis of chronic obstructive pulmonary disease at any time in the study period were excluded.

### Study end points

The primary objective of this analysis was to evaluate the frequency of SMART dosing of BFC based on the dosing instructions written by the health-care provider identified in the CPRD. The next objective was to evaluate the use of SABA rescue medication by examining the number of SABA prescriptions in the year following the index BFC prescription. Adherence to BFC in the SMART and regular maintenance dosing only patients was measured by the proportion of days covered, and the medication possession ratio in a 1-year post index, calculated using prescriptions written and recorded in CPRD.

### Data analysis

Treatment patterns of BFC and SABA were described in the year following the index date to identify SMART dosing instructions that were given with and without rescue medication use. Dosing instructions were captured in CPRD-GOLD when the instructions were within the top 100,000 most common dosing instructions given on any prescription. SMART therapy was identified if the instructions suggested any maintenance dosing regimen with additional dosing instructions for ‘and when required’ (reliever) use (e.g., inhale one puff twice a day, and as needed for symptoms). In determining SMART dosing, this study did not characterise off-label dosing of ICS strengths or maintenance dosing different than twice daily.

Data were analysed descriptively, and no formal statistical comparisons were made. Prescription and patient-level summaries of SMART dosing were tabulated. Patient demographics were described based on the age and gender at the index prescription. Patients were assessed for their use of SABA in the year following the index prescription for BFC. For all analyses, the year following the index date included any BFC prescriptions that were recorded on the index date. Confounders considered in the analysis were age at index date and gender. Prescription- and patient-level data were analysed. To evaluate the potential for time trends, analyses were presented overall and by calendar year of the index date where applicable.

## Figures and Tables

**Table 1:** Age and gender breakdown of patients receiving BFC therapy for asthma on index prescription

*Patient characteristics*	*SMART dosing instructions* *(*N*=173)*		*Maintenance dosing instructions* *(*N*=14,645)*	
	N	*%*	N	*%*
*Age (years)*				
18–25	23	13.3	1,179	8.1
26–44	67	38.7	4,637	31.7
45–64	58	33.5	5,778	39.5
⩾65	25	14.5	3,051	20.8
				
*Gender*				
Female	109	63.0	9,148	62.5
Male	64	37.0	5,497	37.5

Abbreviations: BFC, budesonide/formoterol fixed-dose combination; SMART, single maintenance and reliever therapy.

**Table 2:** Trends in dosing strategy for all BFC prescriptions at prescription and patient level from 2009 to 2013

*Year*	*Prescriptions*					*Patients (according to index prescription)*				
	N	*Maintenance dosing instructions*		*SMART*		N	*Maintenance dosing instructions*		*SMART*	
		n	*%*	N	*%*		N	*%*	N	*%*
2009	27,013	26,820	99.3	193	0.7	6,559	6,505	99.2	54	0.8
2010	27,968	27,759	99.3	209	0.8	2,754	2,723	98.9	31	1.1
2011	30,711	30,398	99.0	313	1.0	3,264	3,208	98.3	56	1.7
2012	22,035	21,771	98.8	264	1.2	2,241	2,209	98.6	32	1.4
2013	14,072	13,897	98.8	175	1.2	0^a^	—	—	—	—
Total	121,799	120,645	99.1	1,154	0.9	14,818	14,645	98.8	173	1.2

Abbreviations: BFC, budesonide/formoterol fixed-dose combination; SMART, single maintenance and reliever therapy.

a One year of follow-up was required; therefore, there are no new (index) prescription patients in 2013.

**Table 3:** SABA (rescue medication) and BFC use by dosing instructions

	*SMART dosing instructions*	*Maintenance dosing instructions*
	N*=173*	N*=14,645*
*SABA in the year following index date (includes index date) n (%):*		
No prescription	82 (47.4)	2,677 (18.3)
Prescription	91 (52.6)	11,968 (81.7)
		
*SABA on the index date n (%)*		
No prescription	146 (84.4)	8,457 (57.8)
Prescription	27 (15.6)	6,188 (42.3)

*Total users of SABA*	N*=91*	N*=11,968*
Mean number of inhalers in year (s.d.)	5.7 (6.0)	5.5 (5.1)
Median	3	4
Min, max^a^	1, 40	1, 64

*Total users of BFC*	N*=173*	N*=14,645*
Mean number of inhalers in year (s.d.)	4.7 (4.3)	4.8 (3.6)
Median	4	4
Min, max^a^	1, 35	1, 36

Abbreviations: BFC, budesonide/formoterol fixed-dose combination; SABA, short-acting β_2_-agonists.

a Note that there are extreme values that are implausible (maximum inhalers), as is noted for electronic records data. The outliers affect both groups and are included to reflect the data.
